# Boosting the Antibacterial Performance of Natural Rubber Latex Foam by Introducing Silver-Doped Zinc Oxide

**DOI:** 10.3390/polym15041040

**Published:** 2023-02-19

**Authors:** Abdulhakim Masa, Nureeyah Jehsoh, Sawitree Dueramae, Nabil Hayeemasae

**Affiliations:** 1 Rubber Engineering & Technology Program, International College, Prince of Songkla University, Songkhla 90110, Thailand; 2 Department of Rubber Technology and Polymer Science, Faculty of Science and Technology, Prince of Songkla University, Pattani Campus, Pattani 94000, Thailand; 3 Division of Biological Science, Faculty of Science, Prince of Songkla University, Songkhla 90110, Thailand

**Keywords:** natural rubber, foam, silver-doped zinc oxide, antibacterial performance

## Abstract

Natural rubber (NR) latex foam is one of the rubber products that are increasingly in demand in the market. This is simply because of its lightweight, good thermal insulation, and resilience. The applications of NR latex foam are mostly for pillows and mattresses. This has resulted in these products requiring antibacterial performance which is very important for the safety of the end-users. In this study, the antibacterial NR latex foam was prepared by incorporating the silver-doped zinc oxide (Ag-doped ZnO) into the NR latex foam. Ag-doped ZnO was prepared by microwave-assisted method and then characterized through morphological characteristics and X-ray diffraction (XRD). The content of Ag doped onto ZnO was designed by varying the AgNO_3_ content at 15 wt%, 50 wt%, and 100 wt% of ZnO. The results confirmed that the Ag was successfully doped onto ZnO. The silver particles were found to be in the 40–50 nm range, where the size of ZnO ranges between 300 and 400 nm, and the Ag attached to the ZnO particles. The XRD patterns of Ag-doped ZnO correspond to planes of hexagonal wurtzite ZnO structure and cubic metallic Ag. This Ag-doped ZnO was further added to NR latex foam. It was observed that Ag-doped ZnO did not affect the physical properties of the NR latex foam. However, it is clear that both the inhibition zone and percent reduction of bacteria (e.g., *E. coli* and *S. aureus*) were enhanced by the addition of Ag-doped ZnO. It showed a decrease in the amount of cell growth over contact time. The content of 100 wt% AgNO_3_ could reduce *E. coli* and *S. aureus* up to 64.72% and 58.90%, respectively, when samples were maintained for 24 h. This study provides a scientific understanding of how Ag-doped ZnO could facilitate the development of eventual rubber foam products based on the respective results.

## 1. Introduction

NR latex is a fascinating bio-polymeric material derived from a tree called *Hevea brasiliensis* or often called rubber tree in our daily lives. In this modern world, this naturally occurring raw material is used to produce a variety of products that make human life easy and comfortable. These include rubber gloves, condoms, catheters, and so forth [1,2]. NR foam is an interesting rubber product that consists of rubber and gas phases in one item. NR foam has been used in many applications because of its lightweight, good thermal insulation, and sound absorption. There are several methods for producing NR foam. Generally, it is produced by the NR latex process, which is in liquid form. Chemical stabilization is necessary since the NR latex is colloidal (usually obtained with the addition of ammonia). The environment and storage period can impact this stabilizing process and the latex quality. In addition to the applications of NR, the need for NR latex foam with antibacterial qualities expanded as consumer health consciousness and the healthcare sector developed. Manufacturers must respond to consumers’ needs by providing antimicrobial solutions in a wide range of applications, from medical devices to construction materials and consumer goods, as consumers are becoming more and more aware of bacteria and their harmful effects, especially in light of the recent COVID-19 pandemic [3,4]. Similarly, the NR-based research area benefited from antimicrobial technology by reducing microbes and increasing the service life of rubber materials.

For decades, metals and metal oxides have been used as antimicrobial agents. Various antimicrobial metal and metal oxide nanoparticles include gold (Au), silver (Ag), aluminum (Al), titanium dioxide (TiO_2_), magnesium oxide (MgO), zinc oxide (ZnO), and copper oxide (CuO) nanoparticles [5,6]. Ag nanoparticles are well-known and prominent antimicrobial metal oxide nanoparticles due to their spectacular properties, as explained below. Ag nanoparticles exhibit high thermal stability and low toxicity to human cells and tissues. Ag nanoparticles are highly toxic to a vast range of pathogenic microbes, and also show long-term activity. Manufacturing polymeric products containing silver nanoparticles that show antimicrobial properties have been a well-known research area among many researchers recently. Polymers modified by Ag nanoparticles have different types of applications, such as antimicrobial, biomedical applications, and applications in catalysts [7,8,9]. ZnO is another metal oxide that has been used as an antibacterial agent in many types of polymers. This is because ZnO enables producing hydrogen peroxide (H_2_O_2_) [10] and reactive oxygen species (ROS), which can interact with the bacteria cells causing cell death [11,12]. When ZnO is in contact with bacteria, it can diffuse through the cell wall and cause the membranes to become disorganized. As a result, there can be a significant buildup of ZnO nanoparticles in the cytoplasm and cell membrane.

Compared to other metal oxides, ZnO nanoparticles can prevent the growth of a broad range of pathogenic bacteria under visible light or dark conditions. Sarih et al. [13] prepared antimicrobial NR latex films with different types of antimicrobial agents (mangosteen peel powder (MPP), ZnO nanoparticles, and povidone-iodine (PVP-I)). The antimicrobial loading was varied from 0.5, to 1.0, and 2.0 parts per hundred rubber (phr) to monitor the effective inhibition of Gram-negative bacteria and fungi growth. For MPP and PVP-I antimicrobial agents, a loading of 2.0 phr showed good antimicrobial efficacy with the largest zone of inhibition. Simultaneously, ZnO nanoparticles demonstrated excellent antimicrobial activity at low concentrations. Li et al. [14] prepared Ag particles loaded on the surface of graphene oxide (GO). When the GO content was only 0.1 phr, the minimum inhibitory concentrations (MIC) of *Escherichia coli* (*E. coli*) and *Staphylococcus aureus* (*S. aureus*) were 16 and 32 μg/mL, respectively. These results are of great significance for improving the barrier and antibacterial properties of medical rubber products.

The ability of ZnO to inhibit the bacteria is mainly from its photocatalytic characteristic. This characteristic can be enhanced by introducing certain methods, such as metal doping [15,16], the creation of heterostructures with semiconductors [17], and the deposition of noble metals [18]. Among these, loading it with noble metals is the most effective method [19,20]. It is possible to increase the photocatalytic activity of ZnO by introducing interfaces between it and noble metals such as Mg, Ag, and Au [21]. This is due to the recombination process being slowed down as a result of photoinduced charges diffusing across the junction between the noble metal and ZnO [22]. Among noble metals, Ag is one of the most desirable noble metals because of its inexpensive price, nontoxicity, and great electrical and thermal conductivity. 

Mechanochemically generated Ag-doped ZnO nanoparticles with different Ag contents were subjected to an antibacterial study against the *E. coli* and *S. aureus* microorganisms [23]. In both instances, it was shown that an increase in the amount of Ag and the resulting reduction in crystallite size increased the zone of inhibition and decreased the minimum inhibitory concentration as well as minimum bactericidal concentration proportionally. In contrast to *S. aureus*, *E. coli* showed this effect more frequently. Based on the proposed mechanisms, it was anticipated that the liberated H_2_O_2_ and the surface contact of Ag with subsequent after-contact effects produced a synergistic action, producing greater antibacterial effectiveness. Rajendran and Mani [24] used Ag-doped ZnO manufactured using a sol-gel technique covered with polyethylene glycol as a stabilizing and capping agent to explore antibacterial activity on many skin bacteria. Due to the inherent antibacterial properties of ZnO and Ag, the maximal was observed. This suggests that Ag-doped ZnO nanoparticles are highly bactericidal toward these skin infections. Recently, Masa et al. [25] incorporated the Ag-doped ZnO into latex films. It was found that Ag-doped ZnO did affect the antibacterial performance against the *E. coli* and *S. aureus* microorganisms and also promoted better reinforcement of the films. From this point of view, it is expected that Ag-doped ZnO could provide a significant effect on other forms of rubber products as well. 

Therefore, this research was performed to prepare an antimicrobial NR latex foam filled with Ag-doped ZnO. Ag-doped ZnO was synthesized using a literature-reported technique. The results obtained from this study will be used for further fabricating antibacterial NR foam products. This is considered a good initiative for preparing antibacterial foam based on Ag-doped ZnO.

## 2. Experimental Details

### 2.1. Materials

The high ammonia centrifuged latex with 60% dry rubber content (60% HA) was purchased from Suansom Kanyang, Yala, Thailand. Silver nitrate (99.9% assay and MW = 169.9 g/mol) was supplied by POCH S.A., Gliwice, Poland. Zinc nitrate hexahydrate (98.0% assay and MW = 297.5 g/mol) and ethylene glycol (99.9% assay and MW = 62.1 g/mol) were distributed by Molecule Co. Ltd., Bangkok, Thailand. Sodium hydroxide (98.0% assay and MW = 40 g/mol) was supplied by Loba Chemie Pvt Ltd., Maharashtra, India. Other additives such as 20% potassium oleate (KOL), 50% zinc diethyl dithiocarbamate (ZDEC), 50% zinc 2-mercaptobenzothiazole (ZMBT), 50% Wingstay L, 50% sulfur, 15% diphenylguanidine (DPG), and 50% sodium silicofluoride (SSF) were supplied by Siamnavakam Co. Ltd., Nonthaburi, Thailand.

### 2.2. Synthesis of ZnO

ZnO particles were prepared using a direct precipitation technique. First, 500 mL of 0.5 M zinc nitrate was mixed with 500 mL of 0.5 M sodium hydroxide under vigorous stirring for 24 h to form a white precipitate. The colloidal solution was filtered, washed, and dried. Finally, the precipitate was calcined at 600 °C with a heating rate of 5 °C/min for 2 h in an ambient atmosphere.

### 2.3. Preparation of Ag-Doped ZnO

**Figure 1:**
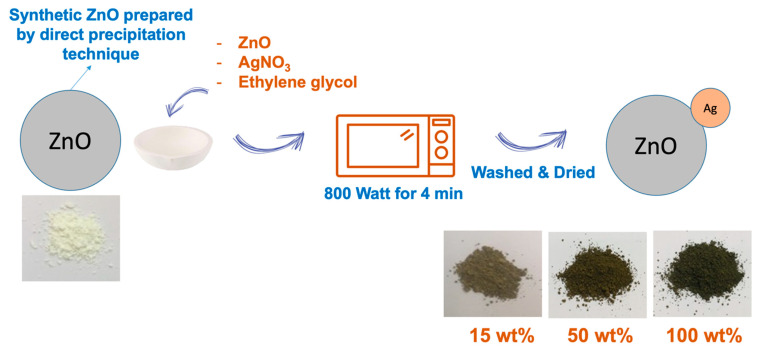
Schematic illustration regarding the steps for synthesis of Ag-doped ZnO.

Figure 1 shows the schematic illustration representing the steps for doping Ag onto ZnO. To prepare Ag-doped ZnO, 2.5 g of ZnO was suspended in 100 mL ethylene glycol under constant stirring for 30 min. AgNO_3_ with 15 wt%, 50 wt%, and 100 wt% of ZnO were varied and added to ZnO suspension. It was then heated in an 800-Watt microwave oven for 4 min. The resultant material was collected, washed with deionized water, and dried for further characterization. Figure 1 also shows the physical appearance of ZnO and Ag-doped ZnO. It was seen that the prepared Ag-doped ZnO has a darker color upon the addition of AgNO_3_. Generally, AgNO_3_ solution is a colorless solution, but the color was changed to yellow when exposed to light. Therefore, the color of ZnO was changed due to the deposition of Ag onto the ZnO.

### 2.4. Characterization of Ag-Doped ZnO

XRD is used to obtain the XRD pattern of the samples. By obtaining the XRD peak pattern, it is easy to identify the crystal structures of ZnO and Ag-doped ZnO. The sample is scanned stepwise in the range of 2θ = 5–80 degree, with a scanning rate of 0.001 degree/s. Fourier transform infrared spectroscopy (FTIR) was used to examine the functionalities shown in ZnO and Ag-doped ZnO via the FTIR spectroscope model TENSOR27. The spectra were captured in transmission mode throughout the range of 4000–550 cm^−1^ at a resolution of 4 cm^−1^ on pellets prepared by 40 times KBr dilution. A transmission electron microscope (TEM) was used. The Cu grid was carefully coated with a little drop of a diluted sample, which was then given 1 h to dry. The Cu grid was then placed inside the apparatus, and the images were captured. An elemental analysis of the rubber sample was screened through energy dispersive spectroscopy equipped with a scanning electron microscope (SEM-EDS).

### 2.5. Preparation of NR Latex Foam Filled with ZnO and Ag-Doped ZnO

Table 1 shows the lab-scale formulation of making pure NR latex foam. The process of making NR latex foam is done by the Dunlop process. First, a certain amount of 60% HA latex was added to the cake beater and the ammonia was slowly evaporated while stirring for 3 min. Next, 20% KOL, 50% ZDEC, 50% ZMBT, 50% Wingstay L, and 50% sulfur were added. Then, the beating speed was increased until the desired volume of foam (5 min) was reached, and 50% DPG and 25% ZnO or Ag-doped ZnO were subsequently added. After that, the gelling agent (20% SSF) was quickly added and beaten for another 1 min. Finally, the ungelled foam was immediately poured into the aluminum mold and allowed to gel for 2 min at ambient temperature. The gelled foam was cured for 2 h at 100 °C under hot steaming. To get rid of soap and unreacted substances, the dried foam was thoroughly cleaned with water after being removed from the mold. The cured NR latex foam was washed, and then dried for 4 h in a hot air oven at 80 °C.

### 2.6. Measurement of Physical Properties

The density of the foam was measured based on the mass per volume. First, the NR foam was also prepared in a square shape with a dimension of 1.0 × 1.0 × 1.0 cm^3^, and the sample was weighed and calculated based on the mass per volume. Compression–deflection test was done according to ASTM D575 [26]. A sample with a dimension of 10.0 × 10.0 × 2.5 cm^3^ was prepared for testing. The specimens were compressed between the parallel metal plates of a universal testing machine (Tinius Olsen, H10KS, Tinius Olsen Ltd., Surrey, UK) until the thickness was reduced by 25%. The reading of the load was taken immediately. The test was repeated with the same specimen until the load readings did not change by more than 5%. The value was recorded in terms of force per area. Compression set was measured according to ASTM D395 [27]. A representative sample with a dimension of 5.0 × 5.0 × 2.5 cm^3^ was used for testing. The test specimens were compressed to 50% of their original thicknesses. The load was released after 70 h and the thickness was measured after 30 min at room temperature. The compression set calculation was as follows.

(1) $$Compression\;set\;\left(\%\right)=\left[\frac{\left(t_{0}-t_{1}\right)}{\left(t_{0}-t_{s}\right)}\right]\times 100$$

where *t*_0_ is the original thickness, *t*_1_ is the thickness of the specimens after the specified recovery period, and *t_s_* is the thickness of the spacer bar used. An average of five replicates from each test was reported.

### 2.7. Antibacterial Study

The antibacterial performance was evaluated by qualitative and quantitative methods. As for the first technique, the NR latex foam with and without Ag-doped ZnO is placed in separate places on Muller-Hinton Agar (MHA) plates in aseptic conditions. The foam was sandwiched between the MHA layers. Furthermore, 15 mL of MHA was added to sterilize the Petri plates under aseptic conditions to create the lower layer. Then, 10 mL of MHA covered the surface of the foam to create the upper thin layer. The optical density (OD) at 600 nm is used to calculate the bacterial concentration. The OD value 0.3 corresponded to the bacteria concentration of 1 × 10^8^ CFU/mL. Amounts of 100 µL of the bacterial solutions of *E. coli* and *S. aureus* were inoculated onto MHA plates and evenly spread. The inoculated agar plates were incubated at 37 °C for 24 h for the inhibition zone measurement.

For quantitative analysis, the test culture was incubated on nutrient agar (NA) medium, incubated at 37 °C for 18–24 h, and single colonies were placed in Muller-Hilton Broth (MHB), and incubated at 37 ± 0.5 °C for 3 h. The turbidity was compared to 0.5 McFarland standard. Furthermore, 100 µL of prepared bacteria was added to 9 mL of MHB medium containing the test samples at different concentrations, e.g., ZnO and Ag-doped ZnO. This was incubated at 37 ± 0.5 °C for 0, 2, 4, 6, 8, and 24 h. Then, the bacteria were diluted 10-fold with 0.85% normal saline, spread onto NA medium, and incubated overnight at 37 ± 0.5 °C. Bacteria survived by a number of colonies (CFU/mL) were counted. The results of the control trials were also recorded to make a comparison. The percent reduction was also reported.

Three replications were implemented for each test. The statistical analysis of the data was performed with the SigmaPlot program (SPSS Inc., IL, USA). Results from individual antibacterial assays were used to construct the statistical difference between the samples with ZnO and Ag-doped ZnO. Differences among treatment groups were examined by using a one-way analysis of variance (disk diffusion test) and a two-way analysis of variance (plate count agar) followed by a Tukey comparisons test. Differences were considered to be statistically significant at *p*-values less than 0.05. 

## 3. Results and Discussion

### 3.1. Characterization of ZnO and Ag-Doped ZnO

**Figure 2:**
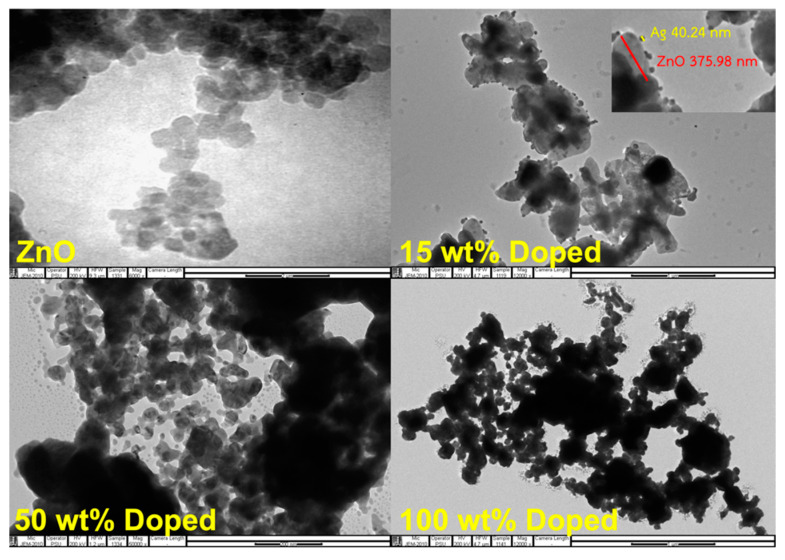
TEM images of ZnO and Ag-doped ZnO at 15–100 wt% of AgNO_3_.

Zinc oxide is one of the metal oxides that can inhibit the growth of bacteria. Research on the use of ZnO as an inhibitor of bacteria has been reported elsewhere. For instance, Liu et al. [28] said that the photocatalytic property of metal oxides is responsible for inhibiting the growth of bacteria. The photocatalytic property can be more effective by doping with other metals. In this work, Ag was doped onto ZnO. The TEM images of synthesized Ag-doped ZnO distributed in distilled water are shown in Figure 2. The particle size measured from the TEM image is also summarized in Table 2. From the photograph, the morphology of two substances can be observed, namely, ZnO and Ag. The morphology of ZnO is shown in gray, while the black color is for Ag. Most particles have sizes between 40 and 50 nm, where the size of ZnO ranges between 300 and 400 nm (see Figure 2). The particles are easily distinguishable. The micrograph contains spherical Ag nanoparticles that are randomly dispersed. 

**Figure 3:**
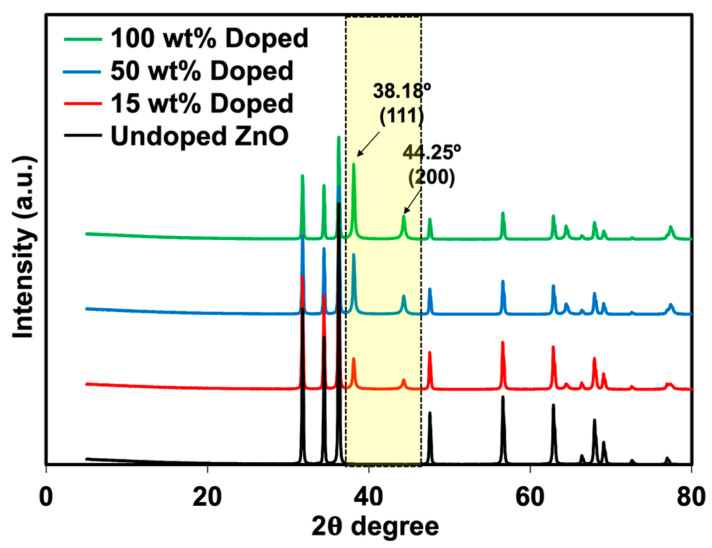
XRD patterns of ZnO and Ag-doped ZnO.

**Figure 4:**
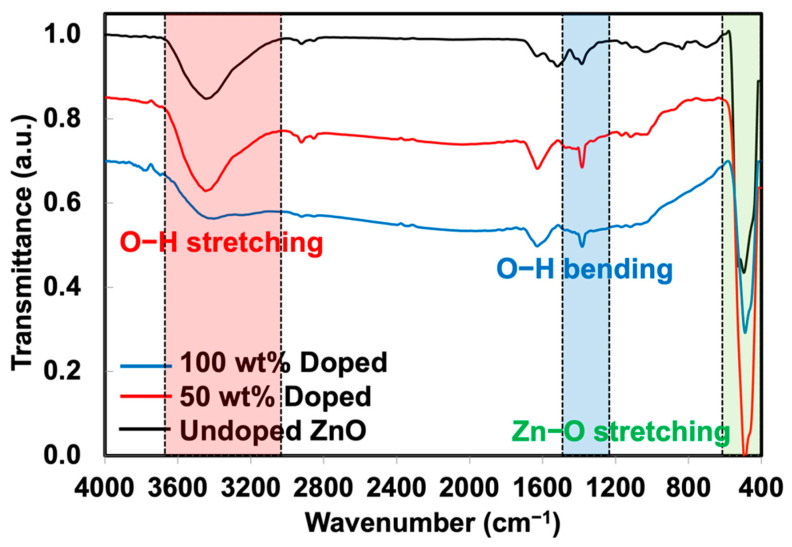
FTIR spectra of ZnO and Ag-doped ZnO.

Phuruangrat et al. [29] prepared the Ag-doped ZnO using the same method and observed that Ag was successfully doped onto ZnO with a size of less than 100 nm. XRD analysis was also carried out to verify the presence of Ag-doped ZnO (see Figure 3). The XRD patterns of these two materials meet the crystallographic library for Ag and ZnO. The crystalline regions of ZnO showed a significant diffraction peak at the 2θ angles of 31.84°, 34.52°, 36.38°, 47.64°, 56.70°, 63.06°, 66.37°, 67.95°, and 69.08° corresponding to (100), (002), (101), (102), (110), (103), (200), (112), and (201) planes of hexagonal wurtzite ZnO structure with reference to JCPDS card No. 01-074-0534. In comparison, the XRD patterns of the Ag-doped ZnO with different Ag loading contents included a major phase of wurtzite ZnO with additional peaks at 2θ angles of 38.18°, 44.25°, and 64.44°, corresponding to (111), (200), and (220) planes of cubic metallic Ag as a minor phase with reference to JCPDS card No. 01-073-6976. This confirms that the doping of Ag onto ZnO was successfully prepared. The results corresponded well with the report by Phuruangrat et al. [29]. The peak intensity of Ag was also increased over the content of AgNO_3_, confirming the presence of higher Ag on ZnO particles. The XRD patterns agreed well with the previous TEM images. The FTIR spectra of ZnO and Ag-doped ZnO are displayed in Figure 4. The stretching mode of the Zn-O bond was attributed to the transmittance found at 480 cm^−1^ [30]. The presence of surface-adsorbed water molecules or hydroxyl groups on the samples is evidenced by the band at 3424 cm^−1^, which is associated with the stretching of the O-H group [31]. The hydroxyl group on the surface of the metal oxide causes a weak reflection at 1380 cm^−1^.

### 3.2. Morphology, Elemental Analysis and Functionalities

**Figure 5:**
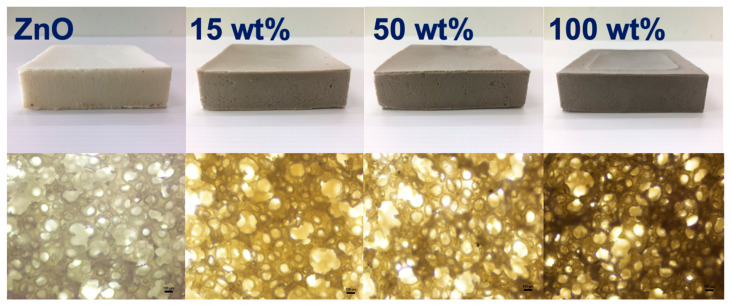
Optical (top) and light microscopic (bottom) images NR foams filled with ZnO and Ag-doped ZnO.

**Figure 6:**
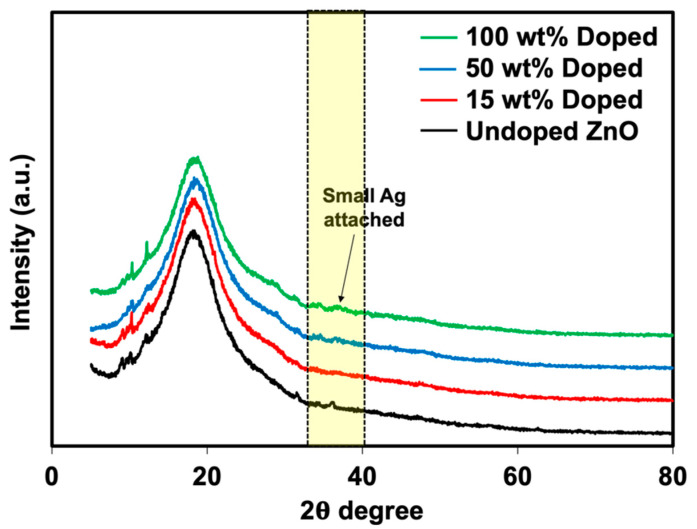
XRD patterns of NR foams filled with ZnO and Ag-doped ZnO.

Figure 5 shows the optical and microscopic images of NR latex foam filled with ZnO and Ag-doped ZnO. It was seen that the prepared foam was darkened due to the AgNO_3_ content. When taking a foamed sample to scan for light microscopy, it was seen that the cell size and structure were almost identical. This is simply due to the content of ZnO that was kept constant at 2 phr. The cell size was in the range of 125–135 µm. Figure 6 shows the XRD patterns of NR latex foams filled with ZnO and Ag-doped ZnO. The peaks were different from previous XRD patterns (see Figure 3). The peak area indicates the amorphous pattern of NR. Adding ZnO and Ag-doped ZnO to the NR latex foam showed a very small difference. It was found that a very minor peak of Ag presented for the sample containing 100 wt% of AgNO_3_ doped onto ZnO. A tiny peak of Ag that appeared may be due to the addition of a small amount of ZnO, e.g., 2 phr, resulting in no apparent crystallization peak of Ag. The presence of Ag-doped ZnO in the NR foam was reconfirmed later by SEM-EDS analysis.

**Figure 7:**
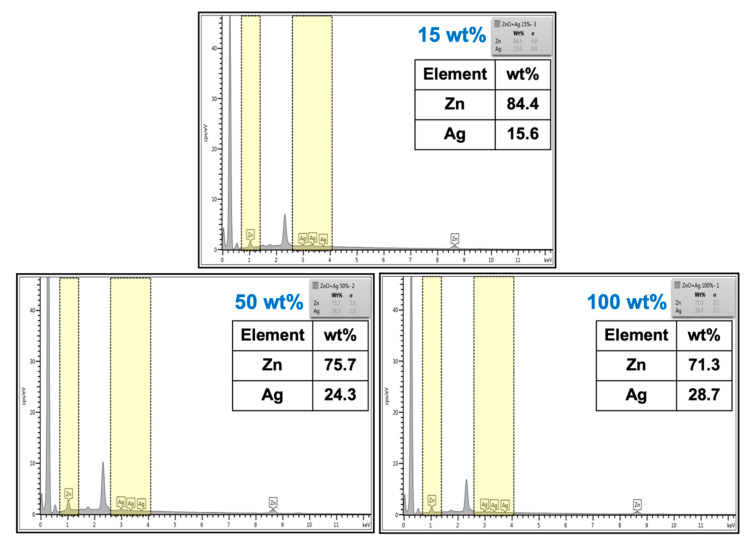
EDS spectrum of NR latex foam filled with Ag-doped ZnO (15 wt%, 50 wt%, and 100 wt% of AgNO_3_).

The characterization of ZnO and Ag-doped ZnO by SEM-EDS is an alternative analysis technique to confirm the presence of elements in the material. Figure 7 shows the SEM images and EDS spectra of NR latex foam filled with Ag-doped ZnO. It is clear that the spectrum of Ag was presented in the EDS results, where the presence of Ag became obvious as the content of AgNO_3_ increased. This confirms the existence of Ag-doped ZnO throughout the NR foam matrix.

### 3.3. Antibacterial Performance

**Figure 8:**
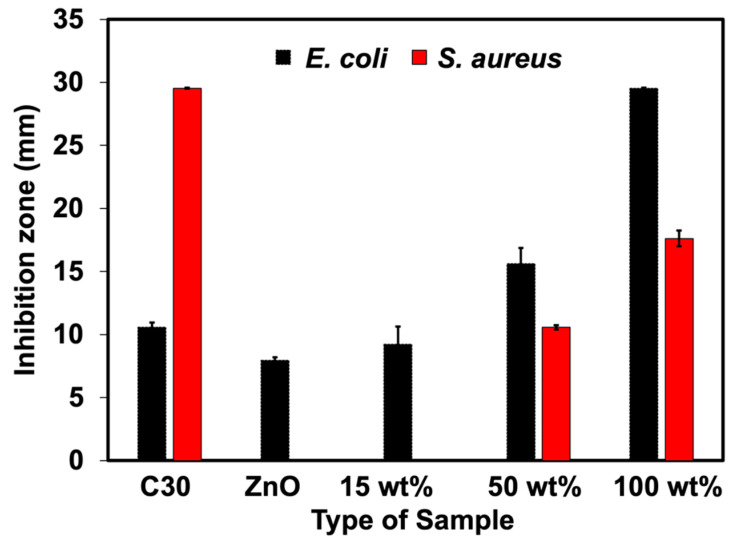
Inhibition zone of NR foams filled with ZnO and Ag-doped ZnO.

**Figure 9:**
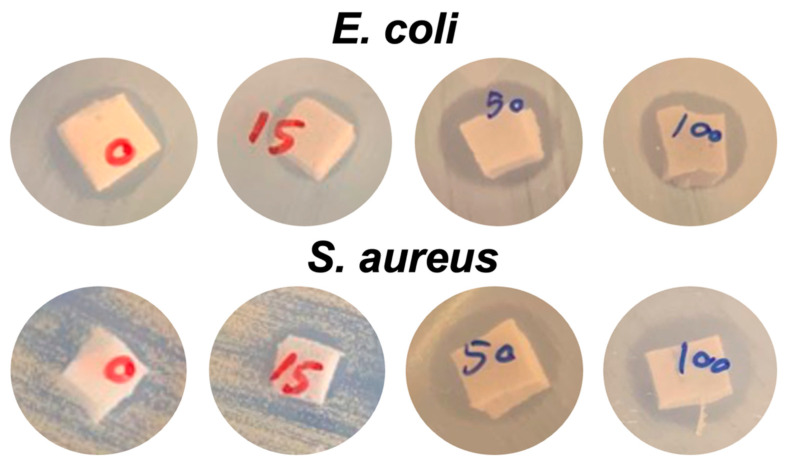
Optical images of the inhibition zone appeared after the antibacterial test of NR foams filled with ZnO and Ag-doped ZnO.

The qualitative results from the disk diffusion test were applied to monitor the antibacterial efficacy of NR latex foam filled with ZnO and Ag-doped ZnO. The measured clear zone and its optical images are illustrated in Figure 8 and Figure 9. The C30 here is the reference antibiotic which is from chloramphenicol. Both types of bacteria, such as *S. aureus* and *E. coli* were inhibited when the ZnO and Ag-doped ZnO was used, the clear zone was found differently when using Ag-doped ZnO at 100 wt% of AgNO_3_. The rate of transmission may depend on the physical characteristics of the culture medium and the type of bacteria. The inhibition of Gram-negative (*E. coli*) was higher than that of Gram-positive (*S. aureus*). The primary cause was anticipated because the peptidoglycan structure of *E. coli* is significantly thinner than that of *S. aureus*, making it much simpler for ZnO or Ag-doped ZnO to enter the cell and kill the *E. coli*. Additionally, a larger concentration of Ag (beyond the threshold limit) can cover the ZnO, which would interfere with the photon capture from the energy source and slow down the photocatalytic action. This is evident in the case of *S. aureus*, where the clear zone was smaller when doped with 100 wt% AgNO_3_. Table 3 shows the raw outputs obtained from statistical analysis. Results showed a statistically significant difference in the antibacterial activity of ZnO and Ag-doped ZnO (*p* < 0.05) against *E. coli* and *S. aureus*. An insignificant difference was observed among the inhibitory activity of ZnO and 15 wt% of AgNO_3_ (both *E. coli* and *S. aureus*). However, the antibacterial potential between ZnO and 50 wt% and 100 wt% of AgNO_3_ showed statistically significant activity in comparison to ZnO (*p* < 0.05).

**Figure 10:**
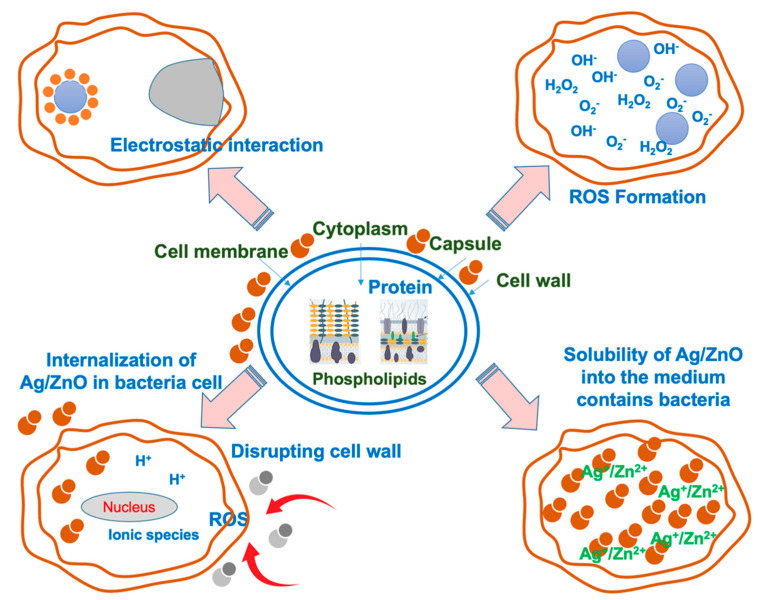
Possible killing mechanisms of NR foams in the presence of ZnO and Ag-doped ZnO.

The possible killing mechanism of bacteria by Ag-doped ZnO can be easily reached by the proposed model shown in Figure 10. It implies that the electron-hole recombination process is minimized by the Ag nanoparticles placed on the surface of ZnO. The tiny Ag clusters that have been formed on the ZnO surface will improve electron transport. ZnO has photocatalytic activity, however, Ag may also possess additional death mechanisms. The proteins found in bacterial cell walls may be denatured by Ag metal ions, inhibiting bacterial growth [32,33]. Additionally, thiol groups and the catalytic oxidation of oxygen molecules in cells are both carried out by Ag ions [34]. Ag accelerates the oxidation process by releasing the active oxygen derivative (ROS), which is comprised of oxygen, hydroxyl, and peroxide free radicals. Zn^2+^ and Ag^+^ are crucial in the demise of the bacteria. This could result in the block of respiration of bacteria. An over-production of ROS, inflammation, and cell cycle disruption are thought to be the primary mechanisms for Ag nanoparticles-induced genetic injuries [35]. Additionally, it has been observed that doping ZnO with metal and metal oxides results in a higher electron-hole charge separation value by lowering the band-gap energy. As a result, the recombination rate decreases, which increases antibacterial activity [11,12].

**Figure 11:**
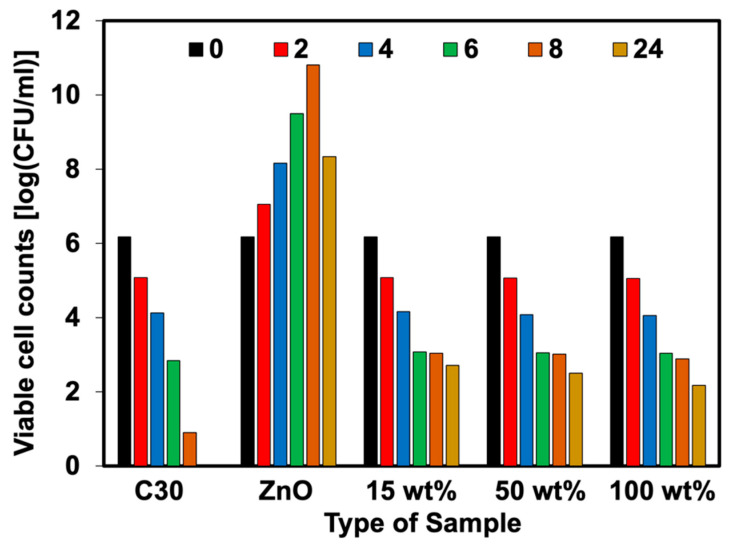
Viable cell counts of *E. coli* against NR foams at various contact times.

**Figure 12:**
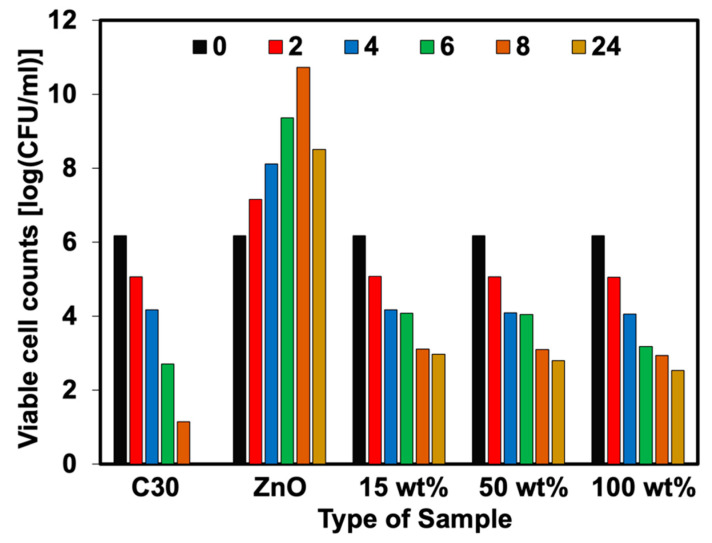
Viable cell counts of *S. aureus* against NR foams at various contact times.

**Figure 13:**
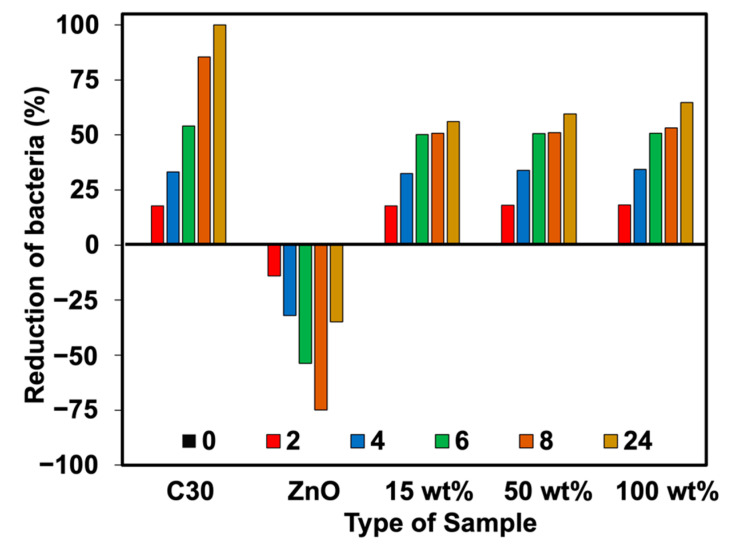
Reduction of *E. coli* against NR foams at various contact times.

**Figure 14:**
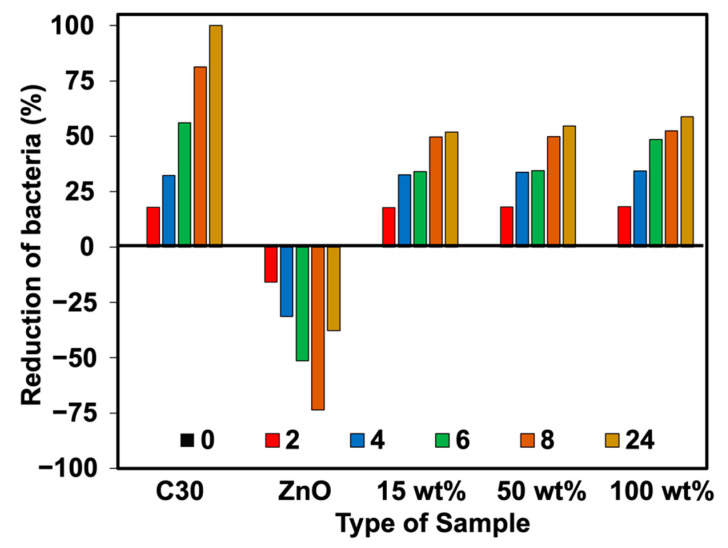
Reduction of *S. aureus* against NR foams at various contact times.

The percent reduction of bacteria (plate count agar) was also analyzed in this work. Figure 11, Figure 12, Figure 13 and Figure 14 show the viable cell counts and their reduction against Gram-negative (*E. coli*) and Gram-positive (*S. aureus*) bacteria. The data were recorded at various timeframes, e.g., 0, 2, 4, 6, 8, and 24 h. It is clear that the survival of bacteria was reduced over the contact time, indicating that higher killing efficiency was obtained over the addition of Ag-doped ZnO. Even though the ZnO is able to kill the bacteria, it does not function well when lasting for longer contact times. It showed an increase in the number of cell growth over the contact time. This has not happened for NR latex foam filled with Ag-doped ZnO. The maximum AgNO_3_ content of 100 wt% could reduce *E. coli* and *S. aureus* up to 64.72% and 58.90%, respectively, when maintaining the samples for 24 h. Table 4 shows the raw outputs obtained from statistical analysis. Results showed a statistically significant difference when considering the antibacterial activity of ZnO and Ag-doped ZnO (*p* < 0.05) against *E. coli* and *S. aureus*. However, an insignificant difference was observed when considering the viable cell count at difference contact times. The viable cell count reduced upon the contact time but with insignificant statistical difference at certain contact time. From qualitative and quantitative analysis, it is clear that Ag-doped ZnO did affect the antibacterial activities, especially at high content of AgNO_3_ (e.g., 50 wt% and 100 wt% of AgNO_3_).

### 3.4. Physical Properties

The foam density, compression–deflection stress, and compression set of NR latex foam filled with ZnO and Ag-doped ZnO are listed in Table 5. It can be seen that the foam density of all samples did not change. General factors affecting the foam density depended on cell size, number of cells, and air phase present in the foam. More air phases can result in a decrease in foam density. In this experiment, the amount of ZnO was kept constant at 2 phr, the only difference is the Ag doping onto the ZnO. Therefore, it did not affect the density of NR latex foam in any way. This corresponds to the compressive stress and compression set of the foams that were observed similarly.

## 4. Conclusions

This work suggested an easy method for preparing Ag-doped ZnO by microwave. It was then incorporated into NR latex foam to perform antibacterial activity. The prepared Ag-doped ZnO was also characterized by certain methods to confirm the presence of Ag on the surface of the ZnO. The Ag nanoparticles were found to be in the 40–50 nm range, where the size of ZnO ranges between 300 and 400 nm. The XRD patterns of Ag-doped ZnO correspond to planes of hexagonal wurtzite ZnO structure (JCPDS card No. 01-074-0534) and cubic metallic Ag (JCPDS card No. 01-073-6976). As for the antibacterial activities, an inhibition zone of bacteria (e.g., *E. coli* and *S. aureus*) was enhanced by adding Ag-doped ZnO. This also reflected the percent reduction of bacteria. Using Ag-doped ZnO significantly affected the reduction of viable cell count. It was observed that Ag-doped ZnO did not affect the physical properties such as foam density, compression set, and compressive stress of the NR foam. Based on the observed findings and corresponding statistical analysis, the use of Ag-doped ZnO at 50 wt% of AgNO_3_ is highly recommended for preparing antibacterial NR latex foam. The results obtained from this study will be useful for further experiments on enhancing antibacterial NR latex foam by applying other key factors. This work is a good initiative for preparing the antibacterial NR foam with a simple fabricating technique.

## Figures and Tables

**Table 1:** Ingredients used for preparing the NR foams.

Ingredients	Quantity (phr)
60% HA	100
20% KOL	2.0
50% ZDEC	1.0
50% ZMBT	1.0
50% Sulfur	2.5
50% Wingstay L	1.5
15% DPG	1.2
25% ZnO or Ag-doped ZnO	2.0
20% SSF	1.2

**Table 2:** Particle size of Ag and ZnO measured by TEM.

AgNO_3_ Content (wt%)	Particle Size (nm)	
	Ag	ZnO
15	47 ± 6	305 ± 40
50	45 ± 4	310 ± 40
100	46 ± 5	300 ± 40

**Table 3:** Comparison of statistical outputs among rubber formulations.

Comparison for Rubber Formulations	*E. coli*		*S. aureus*	
	*p*-Value	*p* < 0.05	*p*-Value	*p* < 0.05
ZnO vs. 15 wt%	0.413	No	1	No
ZnO vs. 50 wt%	<0.001	Yes	<0.001	Yes
ZnO vs. 100 wt%	<0.001	Yes	<0.001	Yes

**Table 4:** Comparison of statistical outputs among rubber formulations and contact times.

**Comparison for Rubber Formulations**	***E. coli***		***S. aureus***	
	***p*-Value**	***p* < 0.05**	***p*-Value**	***p* < 0.05**
ZnO vs. 15 wt%	<0.001	Yes	<0.001	Yes
ZnO vs. 50 wt%	<0.001	Yes	<0.001	Yes
ZnO vs. 100 wt%	<0.001	Yes	<0.001	Yes
**Source of Variation**	***E. coli***		***S. aureus***	
	***p*-Value**	***p* < 0.05**	***p*-Value**	***p* < 0.05**
Formulations	<0.001	Yes	<0.001	Yes
Contact times	0.061	No	0.084	No

**Table 5:** Foam density, compression set, and compressive stress of NR foams filled with ZnO and Ag-doped ZnO.

AgNO_3_ Content (wt%)	Density (g/cm^3^)	Compression Set (%)	Stress at 25% Compression (kPa)
0	0.15 ± 0.01	14.75 ± 0.61	9.85 ± 0.18
15	0.15 ± 0.01	14.75 ± 0.80	10.00 ± 0.25
50	0.15 ± 0.01	14.80 ± 0.91	9.95 ± 0.33
100	0.16 ± 0.01	14.75 ± 0.81	10.50 ± 0.13

## Data Availability

The data presented in this study are available on request from the corresponding author.

## References

[1] Sethulekshmi A.S., Saritha A., Joseph K. (2022). A comprehensive review on the recent advancements in natural rubber nanocomposites. Int. J. Biol. Macromol..

[2] Chen Q., Zhang Z., Huang Y., Zhao H., Chen Z., Gao K., Yue T., Zhang L., Liu J. (2022). Structure-Mechanics Relation of Natural Rubber: Insights from Molecular Dynamics Simulations. ACS Appl. Polym. Mater..

[3] Cesar M.B., Borges F.A., Bilck A.P., Yamashita F., Paulino C.G., Herculano R.D. (2020). Development and Characterization of Natural Rubber Latex and Polylactic Acid Membranes for Biomedical Application. J. Polym. Environ..

[4] Borges F.A., Filho E.d.A., Miranda M.C.R., Santos M.L.d., Herculano R.D., Guastaldi A.C. (2015). Natural rubber latex coated with calcium phosphate for biomedical application. J. Biomater. Sci. Polym. Ed..

[5] Stankic S., Suman S., Haque F., Vidic J. (2016). Pure and multi metal oxide nanoparticles: Synthesis, antibacterial and cytotoxic properties. J. Nanobiotechnol..

[6] Li Y., Zhang W., Niu J., Chen Y. (2012). Mechanism of Photogenerated Reactive Oxygen Species and Correlation with the Antibacterial Properties of Engineered Metal-Oxide Nanoparticles. ACS Nano.

[7] Shuai C., Xu Y., Feng P., Wang G., Xiong S., Peng S. (2019). Antibacterial polymer scaffold based on mesoporous bioactive glass loaded with in situ grown silver. Chem. Eng. J..

[8] Kumar V., Jolivalt C., Pulpytel J., Jafari R., Arefi-Khonsari F. (2013). Development of silver nanoparticle loaded antibacterial polymer mesh using plasma polymerization process. J. Biomed. Mater. Res. Part A.

[9] Jeong S.H., Yeo S.Y., Yi S.C. (2005). The effect of filler particle size on the antibacterial properties of compounded polymer/silver fibers. J. Mater. Sci..

[10] Georgekutty R., Seery M.K., Pillai S.C. (2008). A Highly Efficient Ag-ZnO Photocatalyst: Synthesis, Properties, and Mechanism. J. Phys. Chem. C.

[11] Qi K., Cheng B., Yu J., Ho W. (2017). Review on the improvement of the photocatalytic and antibacterial activities of ZnO. J. Alloys Compd..

[12] Abebe B., Zereffa E.A., Tadesse A., Murth H.C.A. (2020). A Review on Enhancing the Antibacterial Activity of ZnO: Mechanisms and Microscopic Investigation. Nanoscale Res. Lett..

[13] Sarih N.M., Gwee K., Maher S., Rashid A.A. (2022). Natural Rubber (NR) Latex Films with Antimicrobial Properties for Stethoscope Diaphragm Covers. Materials.

[14] Li T., Su Y., Wang D., Mao Y., Wang W., Liu L., Wen S. (2022). High antibacterial and barrier properties of natural rubber comprising of silver-loaded graphene oxide. Int. J. Biol. Macromol..

[15] Park M.-H., Li J.-H., Kumar A., Li G., Yang Y. (2009). Doping of the Metal Oxide Nanostructure and its Influence in Organic Electronics. Adv. Funct. Mater..

[16] Ogale S.B. (2010). Dilute Doping, Defects, and Ferromagnetism in Metal Oxide Systems. Adv. Mater..

[17] Luo Y., Zhang C., Zheng B., Geng X., Debliquy M. (2017). Hydrogen sensors based on noble metal doped metal-oxide semiconductor: A review. Int. J. Hydrogen Energy.

[18] Hämäläinen J., Ritala M., Leskelä M. (2014). Atomic Layer Deposition of Noble Metals and Their Oxides. Chem. Mater..

[19] Mageshwari K., Nataraj D., Pal T., Sathyamoorthy R., Park J. (2015). Improved photocatalytic activity of ZnO coupled CuO nanocomposites synthesized by reflux condensation method. J. Alloys Compd..

[20] Sun L., Zhao D., Song Z., Shan C., Zhang Z., Li B., Shen D. (2011). Gold nanoparticles modified ZnO nanorods with improved photocatalytic activity. J. Colloid Interface Sci..

[21] Etacheri V., Roshan R., Kumar V. (2012). Mg-Doped ZnO Nanoparticles for Efficient Sunlight-Driven Photocatalysis. ACS Appl. Mater. Interfaces.

[22] Wang R., Xin J.H., Yang Y., Liu H., Xu L., Hu J. (2004). The characteristics and photocatalytic activities of silver doped ZnO nanocrystallites. Appl. Surf. Sci..

[23] Talari M.K., Majeed A.B.D., Tripathi D.K., Tripathy M. (2012). Synthesis, characterization and antimicrobial investigation of mechanochemically processed silver doped ZnO nanoparticles. Chem. Pharm. Bull.

[24] Rajendran R., Mani A. (2020). Photocatalytic, antibacterial and anticancer activity of silver-doped zinc oxide nanoparticles. J. Saudi Chem. Soc..

[25] Masa A., Jehsoh N., Saiwari S., Dueramae S., Hayeemasae N. (2023). Microwave-assisted silver-doped zinc oxide towards antibacterial and mechanical performances of natural rubber latex film. Mater. Today Commun..

[26] (2018). Standard Test Methods for Rubber Properties in Compression.

[27] (2018). Standard Test Methods for Rubber Property—Compression Set.

[28] Liu H., Hu Y., Zhang Z., Liu X., Jia H., Xu B. (2015). Synthesis of spherical Ag/ZnO heterostructural composites with excellent photocatalytic activity under visible light and UV irradiation. Appl. Surf. Sci..

[29] Phuruangrat A., Wongwiwat N., Thongtem T., Thongtem S. (2018). Microwave-assisted solution synthesis and photocatalytic activity of Ag nanoparticles supported on ZnO nanostructure flowers. Res. Chem. Intermed..

[30] Srivastava V., Gusain D., Sharma Y.C. (2013). Synthesis, characterization and application of zinc oxide nanoparticles (n-ZnO). Ceram. Int..

[31] Sangeetha G., Rajeshwari S., Venckatesh R. (2011). Green synthesis of zinc oxide nanoparticles by aloe barbadensis miller leaf extract: Structure and optical properties. Mater. Res. Bull..

[32] Nabil H., Ismail H., Azura A.R. (2013). Comparison of thermo-oxidative ageing and thermal analysis of carbon black-filled NR/Virgin EPDM and NR/Recycled EPDM blends. Polym. Test..

[33] Ramesan M.T., Siji C., Kalaprasad G., Bahuleyan B.K., Al-Maghrabi M.A. (2018). Effect of Silver Doped Zinc Oxide as Nanofiller for the Development of Biopolymer Nanocomposites from Chitin and Cashew Gum. J. Polym. Environ..

[34] Li Y., Liao C., Tjong S.C. (2020). Recent Advances in Zinc Oxide Nanostructures with Antimicrobial Activities. Int. J. Mol. Sci..

[35] Agnihotri S., Mukherji S., Mukherji S. (2013). Immobilized silver nanoparticles enhance contact killing and show highest efficacy: Elucidation of the mechanism of bactericidal action of silver. Nanoscale.

